# Management of COVID-19 related tracheal stenosis: The state of art

**DOI:** 10.3389/fsurg.2023.1118477

**Published:** 2023-02-13

**Authors:** Riccardo Orlandi, Federico Raveglia, Matteo Calderoni, Enrico Mario Cassina, Ugo Cioffi, Angelo Guttadauro, Lidia Libretti, Emanuele Pirondini, Arianna Rimessi, Antonio Tuoro, Eliseo Passera

**Affiliations:** ^1^ Department of Thoracic Surgery, University of Milan, Milan, Italy; ^2^ Department of Thoracic Surgery, San Gerardo Hospital, ASST Monza, Monza, Italy; ^3^ Department of Surgery, University of Milan, Milan, Italy; ^4^ Department of Medicine and Surgery, School of Medicine and Surgery, Università degli Studi di Milano Bicocca, Monza, Italy

**Keywords:** tracheal stenosis, thoracic surgery, endoscopic thoracic surgery, COVID-19, tracheal procedures

## Abstract

Tracheal stenosis (TS) is a debilitating disease promoted by pathologic narrowing of the trachea. The acute respiratory distress syndrome caused by COVID-19 has been demonstrated to trigger enhanced inflammatory response and to require prolonged invasive mechanical ventilation as well as high frequency of re-intubation or emergency intubation, thus increasing the rate and complexity of TS. The standard-of-care of COVID-19-related tracheal complications has yet to be established and this is a matter of concern. This review aims at collecting latest evidence on this disease, providing an exhaustive overview on its distinctive features and open issues, and investigating different diagnostic and therapeutic strategies to handle COVID-19-induced TS, focusing on endoscopic versus open surgical approach. The former encompasses bronchoscopic procedures: electrocautery or laser-assisted incisions, ballooning dilation, submucosal steroid injection, endoluminal stenting. The latter consists of tracheal resection with end-to-end anastomosis. As a rule, traditionally, the endoscopic management is restricted to short, low-grade, and simple TS, whereas the open techniques are employed in long, high-grade, and complex TS. However, the critical conditions or extreme comorbidities of several COVID-19 patients, as well as the marked inflammation in tracheal mucosa, have led some authors to apply endoscopic management also in complex TS, recording acceptable results. Although severe COVID-19 seems to be an issue of the past, its long-term complications are still unknown and considering the increased rate and complexity of TS in these patients, we strongly believe that it is worth to focus on it, attempting to find the best management strategy for COVID-19-related TS.

## Introduction

Tracheal stenosis (TS) is an invalidating disease characterized by emphasized tissue fibrotic reaction leading to pathologic narrowing of the trachea (1). Traditionally, iatrogenic intubation injury, prolonged intubation or tracheostomy are the most frequent causes of TS (2). Other causes include local radiotherapy, inflammatory or autoimmune diseases, idiopathic, and neoplastic diseases. Coronavirus Disease 2019 (COVID-19) has been reported to be strictly related to TS. Indeed, longer times of invasive ventilation and differed tracheostomy to promote prone position as well as higher rates of iatrogenic intubation injuries have resulted in significant rise in rate and complexity of TS in patients with severe COVID-19 (3). Experiences on COVID-19-related TS are still limited and there is lack of publications in literature on this topic: its best management has not been yet clearly established. Therefore, we performed a review to provide a brief but exhaustive overview on this relatively new disease, focusing on its distinctive features and open issues.

## Epidemiology

Rate of TS following endotracheal intubation is estimated from 10% to 22%, but only 1%–2% of cases will complain severe dyspnea (4, 5), with reported incidence of 4.9 cases per million per year (6). During the pandemic, almost half of COVID-19 patients in ICU required invasive mechanical ventilation, with mean duration of 17 days and high rate of reintubation (7). The exact number of intubated COVID-19 patients developing TS is unknown, but its incidence is reported to widely range from 3.3% (8) to 40% (9, 10). The raw incidence of TS seems to be higher in COVID-19 patients than in pre-COVID-19 era (11), and the medical community has been alerted to the possibility of an unprecedented surge in TS (12), despite the limited number of cases in literature does not allow to draw definitive conclusions in this regard. The mean age ranges from 50 (13–15) to 60 (8, 11, 16) years old, and males seem to be more prone to develop this disease, with approximately prevalence of 60%–70% (8, 11, 13, 14, 16, 17).

## Risk factors

Risk factors could be divided into patient-related versus mechanical. The formers include patient's comorbidities, poor health conditions, history of diabetes, lower levels of PaO_2_/FiO_2_ and increased hypoxia (10), obesity, severe inflammation as well as microbial dysregulation of the airways (11, 16), COVID-19-related laryngitis and laryngeal edema (14), disrupted laryngotracheal microcirculation due to prothrombotic and antifibrinolytic state, high viral replication in the tracheal epithelium (11) leading to viral tracheitis (18). Instead, the latters consist of iatrogenic lesions during intubation due to poor visibility and chaotic situations, cuff overinflation to avoid aerosol sprays, prolonged intubation, high rate of reintubation, prone position ventilation with movement of the tube, delayed tracheostomy to allow prone position and viral clearance (11), vasopressor use (8), high-dose corticosteroid resulting in mucosal atrophy and altered healing, impaired nursing service due to workload of pandemics.

## Pathophysiology

TS is a pathological narrowing of the trachea, typically occurring in the upper half, at the cuff or, less frequently, at the stomal site, because of mispositioning of tracheostomy or high placement of endotracheal tube. The extension is variable. In 1965 Cooper and Grillo (19) explained the reason, by performing tracheal autoptic evaluation on 30 patients who died during invasive ventilation. The macro and microscopic examination revealed a pattern of damage to the tracheal wall at the cuff site: mucosal hemorrhage or ulceration, necrosis and dissolution of adjacent cartilaginous rings, up to scarring fibrosis. Pathophysiologic basis of this phenomenon must be searched in pressure of the cuff. Cuff pressure of endotracheal tube above the capillary perfusion pressure of tracheal mucosa ranging from 20 to 30 mmHg leads to mucosal ischemia, which, if prolonged, results in submucosal damages (20). Ongoing compression causes injury to cartilaginous rings, which are usually fed by diffusion from mucosa and sub-mucosa. Once full epithelium's depth is interested by ischemic injury, healing can no longer rely on epithelium regeneration, but it requires collagen deposition, leading to scarring (21). On the other hand, the examination of tracheal wall at the stomal site revealed granulation tissue along the stoma and the loss of support of anterior cartilaginous arch because of too large stoma or enlargement by leverage of endotracheal tube (19). Ultimately, whatever the origin, TS is mediated by pathologic deposition of collagen in the upper airway, which is triggered by fibroblasts: several cytokines have been reported to promote the profibrotic myofibroblast phenotype observed in TS (22), such as interleukin IL-1, IL-6, IL-13, fibroblast growth factor (FGF), tumor necrosis factor α (TNF-α) and transforming growth factor β (TGF-β). All these cytokines have been demonstrated to be elicited by COVID-19 (23). Recently, some Authors (11, 15) have described microscopic findings of tracheal samples in COVID-19 patients affected by TS, highlighting patchy coagulative necrosis of the epithelium, granulation tissue, extensive presence of lymphocytes, multinucleated giant cells, non-fistulized abscesses leading to cartilage lysis, as well as thrombotic vessels and lymphocytic microvasculitis. Interestingly, cells with viral cytopathic involvement have been identified (11), and viral particles in tracheal epithelial cells have been detected (24), which could support the pathogenetic role of viral tracheitis theorized by Ershadi and colleagues (18). Moreover, Roncati and colleagues (25) have recently reported, by immunohistochemistry, a high density of IgG4-secreting plasma cells on fibrotic tissue from resected tracheal samples in patients affected by COVID-19-related TS. According to Stratakos and colleagues (16), severe COVID-19 could trigger massive Th2 response, which has already been demonstrated to mediate development of TS (22), by inducing localized IgG4 overproduction with resulting fibrosis and scarring in the upper airway.

## Manifestations

Symptoms can widely vary depending on site and extension of airway stenosis (8): occlusive TS localized within 2–3 cm from vocal cords will occur during the hospitalization with difficulties in extubating the patient or weaning tracheostomy; whereas progressive TS localized beyond 3 cm from vocal cords will show up with progressive shortness of breath after discharging. Manifestations will also depend on the degree of obstruction, which in turn influences airflow rate and pressure changes (8, 17): mild stenosis (<50%) will be broadly asymptomatic since the pressure at the stenosis is comparable to that at the glottic plane; moderate stenosis (51%–70%) will result in fluctuating symptoms, based on significant pressure drop manifesting under exertion or other contexts requiring higher airflow rate; severe stenosis (>71%) will produce symptoms even at rest, due to massive pressure drop. The typical presentation consists of dyspnea, wheezing and stridor on physical examination occurring after several days from extubating (8, 9, 14). Symptoms may include dysphonia and communication difficulties (22), hoarseness, dry cough, and swallowing problems (12). Symptoms can occur immediately after extubating or they can be delayed up to 6 months or even within a few years from ventilation weaning (26). TS at the stomal site could be more subtle and less rapidly progressive, leading to functional impairment up to years or even decades later (8). COVID-19-related TS has more severe clinical presentations than other TS because of more complex stenotic airway segments (11) and delayed diagnosis, since symptoms of TS could be initially misdiagnosed as post-COVID-19 respiratory symptoms (16, 17). Whatever the symptoms, without proper management, TS can lead to life-threatening situations due to impaired respiratory function (22).

## Diagnosis

Clinically, TS are divided into simple (or web-like) and complex stenosis: the formers are less than 1 cm long circumferential stenosis, without any cartilage involvement; the latters are more than 1 cm long articulated and mixed stenosis, with involvement of cartilage (17). TS could potentially affect each segment of the trachea. The majority of COVID-19-related TS is complex (8, 13, 16), with higher incidence of associated tracheomalacia, vocal cord paralysis and tracheoesophageal fistula (13), and localized in the upper third segment (8, 13, 14, 17). The stenotic segment is highly variable in extension, but it is most reported as around 2 cm long (8, 11, 13, 16). The most critical factor in TS is assessing tracheal width in the stenotic segment, which is graded according to Cotton-Myer Classification System, based on endoscopic tracheal evaluation (27): I, cross sectional area (CSA) obstruction <50%; II, CSA obstruction 51%–70%; III, CSA obstruction 71%–99%; IV complete obstruction without detectable lumen. Most patients with COVID-19-related TS are graded III (8, 13, 14). Appropriate diagnostic investigations are mandatory to properly classify the TS and to subsequently tailor the best treatment option for each patient: an accurate physical examination, a thorough radiologic assessment and an extensive endoscopic evaluation are the pillars of an adequate preoperative workup (12). The use of laryngoscopy or flexible bronchoscopy is of utmost importance, enabling to gather several dynamic details on vocal mobility, swallowing function, local inflammation, localization, extent and degree of TS, presence of airway lesions, malacic or scar tissue. Endoscopy is the gold standard and should always be performed. CT scan (more rarely MRI) of the trachea can be highly relevant and complementary, mainly in case of complete TS obstruction, providing the possibility to measure extent and narrowing of TS, or in case of suspected laryngo-tracheal framework alteration. In case of apparent TS, a thorough airway evaluation under general anesthesia should be considered. Pulmonary functions should be multidisciplinary assessed, through routine lung function tests, differentiating TS from other respiratory diseases (12).

## Treatment

The optimal management of TS is still a matter of debate, irrespective of the disease which led to invasive ventilation, and clear guidelines on the best treatment option are still demanded, even more so in COVID-19-related TS. Table 1 gathers main case-series studies available in literature on this topic. Currently, treatment of this disease must be personalized, based on patient's clinical conditions and morbidities, and on anatomic characteristics of the stenotic segment. An early specialistic referral and thorough clinical, radiologic, and endoscopic evaluation are mandatory to plan the best therapeutic management. Main treatment options are endoscopic versus surgical procedures. As a rule, endoscopic approach is recommended as first-line definitive treatment in shorter than 2 cm, low-graded intrinsic and well localized TS, or in heavily comorbid patients on poor general conditions, otherwise it is suggested as bridge therapy for definitive surgery. On the other hand, surgery is recommended in longer than 2 cm, high-graded, complex TS, extended to different segments or associated with malacia, altered laryngo-tracheal framework, as well as in case of unsuccessful multiple endoscopic attempts (12). Another available therapeutic option is the tracheostomy, which should be reserved to selected cases. Indeed, the right planning of the treatment procedure would avoid a tracheostomy or redo-tracheostomy with further tracheal damages; anyway, if needed, tracheostomy should be performed into the stenotic or affected segment not to injury healthy tracheal segments (12), through either open or percutaneous techniques, since they have shown to have comparable results in terms of perioperative mortality and morbidity rates in general population (34), and there are no current evidences on the preferable approach to adopt in COVID-19 patients (35). Eventually, treatment options for COVID-19-related TS are like those adopted for any-causes TS (31).

**Table 1:** Main studies reporting the management of COVID-19-related tracheal stenosis.

References	N^a^ of patients	Type of TS	MC grade	Lenght	Treatment	Technique	Complications	Outcomes
Palacios (13)	63	NA	IV 1 III 56 II 3 I 3	3.5	Endoscopic 0	-	-	-
					Surgical 63	Tracheal resection/anastomosis 59 T-tube placement 1 Tracheostomy 3	Infection 18 Dehiscence 4 T-tube obstruction 7 Restenosis 6 Bleeding 2 Pneumonia 2	73% success
Stratakos (16)	23	Simple 2 Complex 21	III 23	2.85 ± 0.9	Endoscopic 15	Dilation/ablation 3 Dilation/ablation + stenting 12	Pneumothorax 1 Restenosis 7 Stent migration 1	87% success
					Surgical 8	Tracheal resection/anastomosis	Restenosis 2	80% success
Piazza (14)	14	NA	III 14	NA	Endoscopic 0	-	-	-
					Surgical 8	Tracheal resection/anastomosis 8	Restenosis 2	80% success
Piazza (14)	14	NA	III 14	NA	Endoscopic 0	-	-	-
					Surgical 14^b^	Tracheal resection/anastomosis 14	Subcutaneous emphysema 1	93% success
Tintinago (11)	12	Complex 12	II 8 I 4	3.5	Endoscopic 0	-	-	-
					Surgical 11^a^	Tracheal resection/anastomosis 11	Restenosis 1	83% success
Topolnitskiy (28)	11	Simple 5 Complex 6	I 1 II 3 III 6 IV 1	3.4 ± 1.1	Endoscopic 1	Stenting 1	Stent migration 1	0% success
					Surgical 10	Tracheal resection/anastomosis 4 Laryngotracheoplasty 6	Partial dehiscence 1 Synechiae 1	90% success
Onorati (15)	9	NA	NA	NA	Endoscopic 8	Dilation/ablation 4 Dilation + stenting 2 Conservative 2	Restenosis 2	75% success
					Surgical 1^b^	Tracheal resection/anastomosis 1	None	100% success
Ayten (8)	7	Simple 3 Complex 4	III 4 II 2 I 1	1.81 ± 0.8	Endoscopic 3	Dilation 3	None	100% success
					Surgical 4^b^	Tracheal resection/anastomosis 4	None	100% success
Beyoglu (17)	7	NA	III 5 II 2	2.03 ± 0.3	Endoscopic 0	-	-	-
					Surgical 7^b^	Tracheal resection/anastomosis 7	Infection 2 Pneumonia 2 Arrhytmia 1 Anastomotic granulation 1	100% success
Vasudevan (29)	4	NA	NA	NA	Endoscopic 4	Dilation/ablation 2 Conservative 2	Restenosis 3	25% success
					Surgical 0	-	-	-
Tapias (30)	4	NA	NA	2.75	Endoscopic 0	-	-	-
					Surgical 4	Tracheal resection/anastomosis 4	None	100% success
Alturk (31)	2	NA	III 1 II 1	2.7	Endoscopic 0	-	-	-
					Surgical 2^b^	Tracheal resection/anastomosis 2	None	100% success
Gervasio (9)	2	NA	III 1 II 1	NA	Endoscopic 1	Conservative 1	None	100% success
					Surgical 1	Tracheal resection/anastomosis 1	None	100% success
Miwa (32)	2	Simple 2	III 2	NA	Endoscopic 0	-	-	-
					Surgical 2	Tracheostomy 2	None	100% success
Menna (33)	1	Complex 1	III 1	NA	Endoscopic 0	-	-	-
					Surgical 1^b^	Total tracheal replacement 1^c^	Pneumonia 1	100% success

NA, not available information; TS, tracheal stenosis; MC, Myer-Cotton.

^a^ 1 patient died before the procedure.

^b^ After endoscopic dilation bridge.

^c^ Cryopreserved aortic allograft.

## Conservative treatment

Conservative procedures consist of rigid bronchoscopy with tracheal dilation by ballooning or mucosal resection through electrocautery, cryoablation or laser, resection of granulation tissue, stenting, intralesional mucosal steroid injection (12). The best endoscopic technique, which could be variably combined, depends on experience of the center and on type of TS. Onorati and colleagues (15) favor endoscopic treatment in COVID-19-related TS, mainly in case of persistent local inflammation of the trachea, reporting encouraging results through bronchoscopic procedures (balloon dilation, stenting, and resection of granuloma) on 8 patients, with 75% success and 25% complication rates. Ayten and colleagues (8) showed 100% success rate in patients affected by simple TS undergoing 1–3 bronchoscopy dilation procedures; they suggest applying endoscopic procedures as first-line therapy in web-like TS smaller than 1 cm and without malacia. Mattioli and colleagues (3) suggest to avoide surgery as primary choice in COVID-19 patients, because of their heavy comorbidities and debilitated conditions, proposing to use balloon dilation procedures with intralesional corticosteroid injection, even in case of complex TS, particularly in “young and thin” stenosis, allowing to heal some patients, or at least to buy time delaying surgery to gain better clinical conditions. Indeed, according to authors supporting conservative treatments, surgical procedures should be reserved to selected fit patients without significative comorbidities (3). Stratakos and colleagues (16) reported 88% success rate after endoscopic dilation and silicon stenting in 15 patients, with 60% complication rate mainly related to stent obstruction due to mucous accumulation and pseudomembranous. Tracheal resection and anastomosis are contraindicated in TS longer than 5–6 cm (8, 14, 31), because of the marked increase in anastomotic complications, and in such contexts, endoscopy could be considered with salvage intent before performing definitive tracheostomy or Montgomery T-tube placement. Since patients could relapse after endoscopic management, it is advisable to strictly follow-up them: they are thought to require an overage of 3.5 review flexible bronchoscopy procedures within the first 6 months (16). Medical therapy with antibiotics and intravenous steroid injection can be considered as ancillary: some authors (9, 15) reported clinical improvement and successful discharge of patients without further need for invasive procedures.

## Surgical treatment

Surgical approaches include right thoracotomy, cervicotomy, cervical collar T-incision with or without manubrium split, median sternotomy, depending on experience of the center and localization of the stenotic segment. Whatever the chosen approach, the tracheal stenotic segment must be released, the damaged rings are removed, then end-to-end anastomosis is performed with 3–0 monofilament sutures by continuous as well as interrupted sutures (36). Traditionally, tracheal resection with end-to-end anastomosis is considered the gold standard treatment in TS (11), even if there are no specific guidelines. Several authors (8, 11–14, 17, 31) believe that surgical treatment is the standard of care also in COVID-19-related TS, especially in complex and articulated stenosis with cartilaginous involvement. In these cases, some authors (8, 13) consider endoscopic procedures even contraindicated, since the high rate of recurrence and the potential increase of the injured segment could decrease the chance of successful surgery. Others (11, 14, 31) rather suggest performing bronchoscopic dilation preoperatively in symptomatic patients as a bridge to definitive surgery, also in reiterated sessions. Concerning the timing of surgery, prevailing indication is to repair TS as early as possible, after the patient has tested negative for SARS-CoV-2 and as soon as he has recovered from the hospitalization (11, 17), if local inflammation is off and chronic steroid course can be discontinued to avoid anastomotic healing complications (15). Beyoglu and colleagues (17) propose to consider the time elapsed between the dilating procedure and the recurrence of symptoms to choose the right timing for surgical procedure: surgery should be planned if the time span between two consecutive sessions is less than 2 weeks. Different studies have reported complication rate of 15%–45% after tracheal resection and anastomosis for any-causes TS (37). Regarding COVID-19-related TS, complication rates range from less than 15% (8, 14) to over 40% (13, 17), whereas the reported success rate is around 80% (11, 13, 16), with 30-day mortality rate of 0%. In case of TS recurrence after surgery, which is reported to occur in 10%–20% of patients (13, 14, 16), dilating rigid bronchoscopy and eventually stenting is indicated as second-line treatments; in case of further failure definitive tracheostomy or Montgomery T-tube placement must be considered.

## Prevention

To prevent COVID-19-related TS, the most critical element is to carefully manage mechanical risk factors listed above, especially choosing the appropriate endotracheal tube for each patient and strictly monitoring cuff pressure (17). The quality of care in COVID-19 has significantly improved since start of the pandemic, and this should slow down the presumed increase in TS rate. Anyway, an early diagnosis is of utmost importance, and therefore each patient with medical history of COVID-19-related invasive ventilation should be clinically, radiologically, and eventually endoscopically followed up to early detect any signs of TS (10, 12), suspecting TS in case of breathing distress after mechanical ventilation weaning (31). Topical or systemic use of steroids, as well as antibiotics and anti-inflammatory drugs, together with early endoscopic dilation and local debridement, could avoid progression to major TS (12).

## Conclusion

COVID-19-related TS may become a relevant pathology within the next few years: it has distinctive features which differentiate it from other-causes TS, and it is worth to hold attention on its development. An early diagnosis is fundamental, and it is based on clinical, radiological, and endoscopic investigations. Patients diagnosed with COVID-19-related TS should be referred to experienced tertiary centers. Treatment should be personalized and tailored on each patient, through multidisciplinary discussion. Therapeutic options consist of endoscopic or surgical procedures, which could provide high success and low complication rates when performed on selected patient in right timing.
